# Identification of aberrantly methylated differentially expressed genes and pro-tumorigenic role of KIF2C in melanoma

**DOI:** 10.3389/fgene.2022.817656

**Published:** 2022-07-22

**Authors:** Chun-Hui Huang, Wei Han, Yi-Zhu Wu, Guo-Liang Shen

**Affiliations:** ^1^ Department of Burn and Plastic Surgery, The First Affiliated Hospital of Soochow University, Suzhou, China; ^2^ Department of Surgery, Soochow University, Suzhou, China; ^3^ Institute of Regenerative Biology and Medicine, Helmholtz Zentrum München, Munich, Germany

**Keywords:** melanoma, methylation, hub genes, prognosis, nevus, KIF2C

## Abstract

**Background:** Skin Cutaneous Melanoma (SKCM) is known as an aggressive malignant cancer, which could be directly derived from melanocytic nevi. However, the molecular mechanisms underlying the malignant transformation of melanocytes and melanoma tumor progression still remain unclear. Increasing research showed significant roles of epigenetic modifications, especially DNA methylation, in melanoma. This study focused on the identification and analysis of methylation-regulated differentially expressed genes (MeDEGs) between melanocytic nevus and malignant melanoma in genome-wide profiles.

**Methods:** The gene expression profiling datasets (GSE3189 and GSE114445) and gene methylation profiling datasets (GSE86355 and GSE120878) were downloaded from the Gene Expression Omnibus (GEO) database. Differentially expressed genes (DEGs) and differentially methylated genes (DMGs) were identified via GEO2R. MeDEGs were obtained by integrating the DEGs and DMGs. Then, a functional enrichment analysis of MeDEGs was performed. STRING and Cytoscape were used to describe the protein-protein interaction (PPI) network. Furthermore, survival analysis was implemented to select the prognostic hub genes. Next, we conducted gene set enrichment analysis (GSEA) of hub genes. To validate, SKCM cell culture and lentivirus infection was performed to reveal the expression and behavior pattern of KIF2C. Patients and specimens were collected and then immunohistochemistry (IHC) staining was conducted.

**Results:** We identified 237 hypomethylated, upregulated genes and 182 hypermethylated, downregulated genes. Hypomethylation-upregulated genes were enriched in biological processes of the oxidation-reduction process, cell proliferation, cell division, phosphorylation, extracellular matrix disassembly and protein sumoylation. Pathway enrichment showed selenocompound metabolism, small cell lung cancer and lysosome. Hypermethylation-downregulated genes were enriched in biological processes of positive regulation of transcription from RNA polymerase II promoter, cell adhesion, cell proliferation, positive regulation of transcription, DNA-templated and angiogenesis. The most significantly enriched pathways involved the transcriptional misregulation in cancer, circadian rhythm, tight junction, protein digestion and absorption and Hippo signaling pathway. After PPI establishment and survival analysis, seven prognostic hub genes were CKS2, DTL, KIF2C, KPNA2, MYBL2, TPX2, and FBL. Moreover, the most involved hallmarks obtained by GSEA were E2F targets, G2M checkpoint and mitotic spindle. Importantly, among the 7 hub genes, we found that down-regulated level of KIF2C expression significantly inhibited the proliferative ability of SKCM cells and suppressed the metastasis capacity of SKCM cells.

**Conclusions:** Our study identified potential aberrantly methylated-differentially expressed genes participating in the process of malignant transformation from nevus to melanoma tissues based on comprehensive genomic profiles. Transcription profiles of CKS2, DTL, KIF2C, KPNA2, MYBL2, TPX2, and FBL provided clues of aberrantly methylation-based biomarkers, which might improve the development of precision medicine. KIF2C plays a pro-tumorigenic role and potentially inhibited the proliferative ability in SKCM.

## Background

Skin cutaneous melanoma (SKCM) is an aggressive tumor that is the fifth and sixth most common malignant tumor in men and women respectively (Siegel et al., 2020). Each year, melanoma accounts for over 80% of skin cancer-related deaths in the world (Schadendorf van Akkooi et al., 2018). According to the Clark model, the pathogenesis of melanoma assumes that numerous steps are required for the progression from melanocytes to malignant melanoma, including the formation of banal nevi, dysplastic nevi, melanoma *in situ*, and invasive melanoma (Wallace et al., 1984). However, the molecular mechanisms underlying the malignant transformation of melanocytes and melanoma tumor progression still remain unclear. Nowadays, as for the primary tumors, surgical resection is usually preferred, while metastatic melanoma is much more difficult to treat with radiotherapy and chemotherapy, which means that early diagnosis is essential (Gadeliya Goodson and Grossman, 2009). Recently developed immunotherapies and targeted therapies show promise for improving the prognosis of patients with advanced melanoma (Flaherty et al., 2012). Identification of melanoma-associated oncogenes informs different therapeutic strategies, and small molecule inhibitors are available to target specific proteins involved in the pathogenesis of melanoma (Mohammadpour et al., 2019). Unfortunately, most patients with melanoma, which are initially diagnosed with highly aggressive and progressive disease, are not candidates for curative therapies (Schadendorf van Akkooi et al., 2018).

DNA methylation is known as a central epigenetic modification, and a significant regulator of gene expression, which can inhibit the binding of transcription factors or the recruitment of repression proteins (Moore et al., 2013). Aberrant promoter methylation of genes that control cell cycle and apoptosis can contribute to the disruption of normal cell division and carcinogenesis (Schinke et al., 2010). Importantly, aberrant DNA methylation is regarded as an epigenetic hallmark of melanoma and plays a significant part in the formation as well as progression of melanoma (Schinke et al., 2010; Goran Micevic et al., 2017). Methylation of CpG islands appears early in tumorigenesis and the epigenetic changes can be identified in serum, sputum, and urine samples which means it might contribute to the development of molecular strategies for cancer detection as well as function as a biomarker of cancer recurrence after excision (James and Herman, 2003). Furthermore, it was reported that hypermethylation correlated with worse prognosis as well as drug resistance (Mori et al., 2005). Increasing evidence showed a vital role for both global hypomethylation of oncogenes and hypermethylation of tumor suppressor genes in tumor development and progression, including in melanoma. For example, methylation silencing of PTEN, an inhibitor of PI3K signaling, was closely related to a worse prognosis in melanoma patients (Lahtz et al., 2010). In addition, the hypermethylation of WIF1, TFPI2, RASSF1A, and SOCS1 has been considered as significant participants in the melanoma initiation and progression (Tanemura et al., 2009). Although research on the identification of separate genes with specific hypermethylation or hypomethylation in SKCM are available, comprehensive network studies based on gene expression, methylation profiles and associated pathways have been greatly insufficient.

Over the last decade, bioinformatics technology has emerged as an indispensable tool for tumor research. It mainly focuses on genomics and proteomics to identify genotypes and phenotypes associated with immune infiltration, tumorigenesis and progression of melanoma to guide the development of targeted therapy (Fan et al., 2018; Zhang et al., 2019). For example, Weiyang Cai et al. (2018) identified many differentially methylated genes (DMGs) related to lymph node metastasis in melanoma and were closely associated with the prognosis of melanoma patients. Duan et al. (2018) found three methylated genes (*ARX*, *DDB2*, and *MBP*) that may be closely associated with the underlying mechanism in melanoma progression. Although methylation changes in SKCM were studied in many research, countless issues are still unclear.

Here, we performed an integrated bioinformatics analysis based on gene expression profiling by high-throughput sequencing (GSE3189 and GSE114445) and gene methylation profiling microarray (GSE86355 and GSE120878). The methylation-regulated differentially expressed genes (MeDEGs) were screened and performed functional enrichment analysis. Furthermore, protein-protein interaction (PPI) networks and survival analysis were used to identify new prognostic biomarkers and therapeutic targets for future research in melanoma.

## Methods

### Acquisition and standardization of raw microarray dataset

We downloaded the gene expression profiling datasets generated by high-throughput sequencing (GSE3189 and GSE114445) and the microarray-based gene methylation profiling datasets (GSE120878 and GSE86355) from the Gene Expression Omnibus database (GEO, https://www.ncbi.nlm.nih.gov/geo/). Totally 18 nevi and 45 melanoma samples were included in GSE3189 (platform: GPL96 Affymetrix Human Genome U133A Array) while 5 nevi and 16 melanoma samples were enrolled in GSE114445 (platform: GPL570 Affymetrix Human Genome U133 Plus 2.0 Array). As for the DNA methylation datasets, GSE120878 included a total of 73 nevi and 89 primary SKCM tissues, while GSE86355 included altogether 14 nevi and 33 primary SKCM tissues. Both of these two methylation microarrays were based on the GPL13534 platform (Illumina HumanMethylation450 BeadChip).

### Identification of methylation-regulated differentially expressed genes

GEO2R (http://www.ncbi.nlm.nih.gov/geo/geo2r/) is a web tool to make a comparison of two or more groups of samples in a GEO Series to screen genes that are differentially expressed across specific experimental conditions. In the present study, GEO2R was used to identify the differentially expressed genes (DEGs) as well as the differentially methylated genes (DMGs). |t|>2 and *p* < 0.05 were considered statistically significant. Then, hypomethylation-high expression genes were obtained after the overlap of upregulated and hypomethylated genes, and hypermethylation-low expression genes were obtained after the overlap of downregulated and hypermethylated genes. The hypomethylation-high expression genes and hypermethylation-low expression genes were identified as methylation-regulated differentially expressed genes (MeDEGs).

### Functional enrichment analysis

The Database for Annotation, Visualization and Integrated Discovery (DAVID, https://david.ncifcrf.gov/) is a straightforward web tool that can provide integrative and systematic annotation for users to unravel biological interactions of multiple genes. It was utilized to perform functional and pathway enrichment analyses. Gene ontology (GO) analysis including the biological process (BP), cellular component (CC), molecular function (MF) and Kyoto Encyclopedia of Genes and Genomes (KEGG) pathway enrichment analysis were conducted for the selected MeDEGs by DAVID (Ashburner et al., 2000; Huang et al., 2007). *p*-value < 0.05 was considered statistically significant.

### Protein-protein interaction network construction and identification of hub genes 

In this study, STRING (http://string-db.org; version 11.0) was adopted to describe protein co-regulation of hypomethylation-high expression genes and hypermethylation-low expression genes respectively and measure functional interactions among nodes (Franceschini et al., 2013). The interaction specificity score >0.4 (the default threshold in the STRING database) was considered statistically significant. Cytoscape (version 3.6.0) was used to visualize interaction networks obtained from STRING (Smoot et al., 2011). MCODE (version 1.4.2) of Cytoscape is a plug-in to cluster a given network to identify densely connected regions based on topology (Bandettini et al., 2012). It was utilized to find the most related module network with selection threshold as follows: MCODE scores >5, degree cutoff=2, node score cut-off=0.2, Max depth=100 and k-score=2.

### Survival analysis

Gene Expression Profiling Interactive Analysis (GEPIA, http://gepia.cancer-pku.cn/) is an online tool that can provide customizable functionalities based on data from The Cancer Genome Atlas (TCGA; https://tcga-data.nci.nih.gov/tcga/) and the Genotype-Tissue Expression project (GTEx; https://www.gtexportal.org/home/index.html) (Tang et al., 2017). GEPIA performs survival analysis based on gene expression levels, using a log-rank test for the hypothesis evaluation. The horizontal axis (x-axis) represented the time in days, and the vertical axis (y-axis) showed the probability of surviving or the proportion of people surviving. The lines presented the survival curves of two groups.

### Validation of hub genes

Oncomine (https://www.oncomine.org) is an online database that allows users to collect, normalize, and analyze gene expression profiling data for tumor samples (Rhodes et al., 2007). Oncomine database was utilized to validate the differential expression of hub genes between SKCM and nevus samples. After choosing the catalog of SKCM and nevus tissue, a comparison of mRNA expression levels was made. The cBioPortal (http://cbioportal.org) is an open-access resource for users to search for multidimensional cancer genomics datasets which provide access to data for over 5000 tumor samples from 20 cancer studies (Cerami et al., 2012). We used cBioPortal to investigate the genetic alterations of hub genes as well as the correlation between methylation status and gene expression.

### Transcription factor network and data processing of gene set enrichment analysis

Transcription factor regulation networks of hub genes were constructed by using R software (Version 3.3.2). Significant nodes involved in co-regulation of *CKS2, DTL, KIF2C, KPNA2, MYBL2, TPX2* and *FBL* were described in circle plots (including transcription factor regulation-DNA binding, related lncRNA, targeted miRNA and protein-protein interaction). Based on data from the TCGA database, GSEA tool (version 2.10.1 package) was used to predict associated up- and down-regulated genes and their significantly involved hallmarks pathways (Subramanian et al., 2005). Student’s-t-test statistical score was implemented in consistent pathways and the mean of the differentially expressed genes was calculated for each analysis. A permutation test with 1000 times was utilized to recognize the greatly involved pathways. The adj. P using Benjamini and Hochberg (BH) and false discovery rate (FDR) method by default were used to correct for the occurrence of false-positive results. Significantly related genes were defined with an adj. *p* < 0.01 and FDR < 0.25.

### Cell culture and lentivirus infection

The human SKCM cell lines (A375) are widely used and representative in various SKCM studies, which were obtained from the Cell Bank of Shanghai Institutes of Biological Sciences, Chinese Academy of Sciences (Shanghai, China). The SKCM cells were cultured in RPMI 1640 medium (Gibco, CA, United States) with 10% fetal bovine serum (Gibco, United States) and 1% penicillin-streptomycin solution (Gibco, CA, United States) at 37°C in a humidified incubator containing 5% CO_2_. The medium was refreshed every 2 days. To establish a stable cell line and intervene the expression of KIF2C, lentiviral vectors and small interfering RNA (siRNA; siRNA-1: 5′-GCC​CAC​TGA​ATA​AGC​AAG​AAT-3'; siRNA-2: 5′-GCC​CGA​ATG​ATT​AAA​GAA​TTT-3′) targeting KIF2C (NM_006845) were obtained from GeneChem (Shanghai, China). Cells were transfected and underwent sterility testing with lentivirus, strictly following the manufacturer’s instructions (GeneChem, China).

### Quantitative reverse transcriptase-polymerase chain reaction

We used TRIzol reagent (Solarbio, China) to extract total RNA from A375 cells, and transcribed them into complementary DNA. Subsequently, SYBR green PCR Master Kit (QIAGEN, Germany) was used for qRT-PCR. The primer sequences for KIF2C and β-actin were designed and displayed in Supplementary Table S1. The reaction conditions of qRT-PCR were conducted according to manufacturers’ protocols (Livak and Schmittgen, 2001) and briefly shown as follows: initial heat activation at 95°C for 2 min and denaturation at 95°C for 5 s, consecutively followed by 40 cycles of 60°C for 30 s and a final extension step. The level of RNA expression was determined by the original Ct value and the 2^−∆∆Ct^ method.

### Patients and specimens collection

All procedures performed in this study involving human participants were in accordance with the Declaration of Helsinki. The study was approved by the ethics committee of the First Affiliated Hospital of Soochow University and informed consent was taken from all the patients. We collected fresh samples from 33 patients at the First Affiliated Hospital of Soochow University from 2019 to 2021. All patients underwent surgeries, had not received preoperative therapy, and were pathologically diagnosed with SKCM. Tissue specimens were obtained during surgery and immediately preserved at −80°C.

### Immunohistochemistry staining

We collected 33 SKCM tissues from patients undergoing surgery at the First Affiliated Hospital of Soochow University. All of the tissues were pathologically confirmed to be SKCM and fixed in 4% paraformaldehyde overnight and embedded in paraffin. Sections were deparaffinized with xylene and hydrated with graded alcohols. Subsequently, the sections were incubated with 3% H_2_O_2_ for 10 min at 37°C and washed in phosphate-buffered saline (PBS). The sections were then incubated with 50 μl of rabbit monoclonal anti-KIF2C antibody (No. ab71706, Abcam, United States) at 4°C overnight. The dilution ratio of the antibody was dependent on the recommended dilution ratio in the specifications. Next, the sections were incubated with PV-6001 (ZSBG, Beijing, China) for 30 min at 25°C. Finally, we stained the slices with 3,3′-diaminobenzidine and hematoxylin for detection. A positive reaction was defined as cytoplasm showing a brown signal. The degree of immunostaining was performed independently by 2 experienced pathologists. The immunostaining score depended on the percentage of positive cells (range: 0–4%; 0, <5%; 1%, 5–25%; 2%, 25–50%; 3%, 51–75%; and 4%, >75%) multiplied by the immunostaining intensity (range: 0–4; 0, non-staining; 1, low intensity; 2 median intensity; and 3, high intensity).

### Cell proliferation and migration assays

The stably transfected A375 were divided into different groups and seeded onto a 96-well plate at a density of 5 × 104 cells/ml. We used the Cell Counting Kit-8 (CCK-8 Kit; Dojindo, Japan), based on the manufacturer’s instructions, to determine the proliferative capacity of cells. Optical density (OD) values were obtained at 450 nm after 24, 48, 72, 96, and 120 h. A transwell cell migration assay was used to test the ability of cells to metastasize. The cell density of different groups was adjusted to 2 × 105 cells/ml, and 100 μl cell suspension of different groups was added to the upper chamber with or without Matrigel (Corning, United States). The cells were cultured for 48 h in a humidified incubator containing 5% CO_2_ at 37°C. The cells were then removed, fixed with 4% paraformaldehyde for 30 min, washed 3 times with PBS, stained with 1% crystal violet for 30 min, and rewashed with PBS. Each sample was viewed and photographed under a microscope in 5 fields. Crystal violet was eluted with 300 μl of 33% acetic acid, and 100 μl cell suspension of different groups was added to each of the 96-well plates. OD value at 450 nm was determined. Migrated cells were imaged in a randomly chosen field of view and counted utilizing the ×200 microscope.

## Results

### Identification of methylation-regulated differentially expressed genes in skin cutaneous melanoma

**FIGURE 1:**
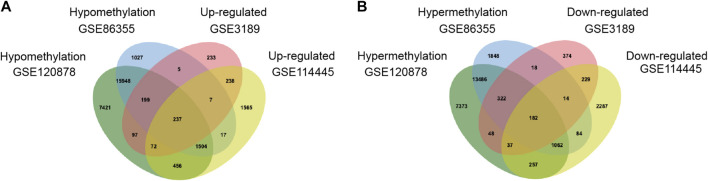
Identification of methylation-regulated differentially expressed genes(MeDEGs) in gene expression datasets (GSE3189 and GSE114445) and gene methylation datasets (GSE86355 and GSE120878). **(A)** hypomethylation and upregulated genes; **(B)** hypermethylation and down-regulated genes.

**FIGURE 2:**
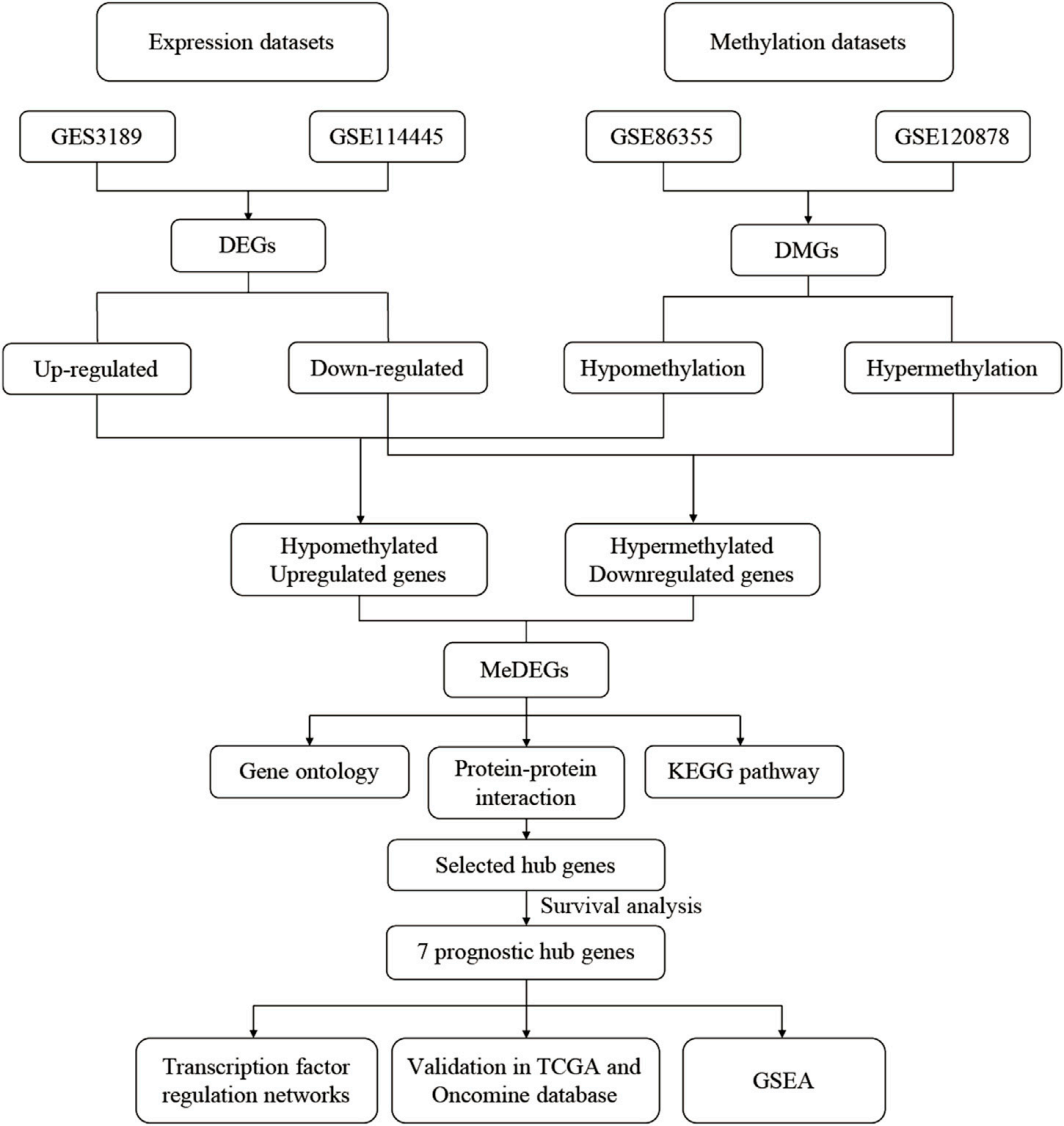
Flowchart of bioinformatics analysis. DEGs, Differentially expressed genes;DMGs, differentially methylated genes; MeDEGs, methylation-regulated differentiallyexpressed genes; KEGG, Kyoto Encyclopedia of Genes and Genomes; GSEA, gene setenrichment analysis.

**FIGURE 3:**
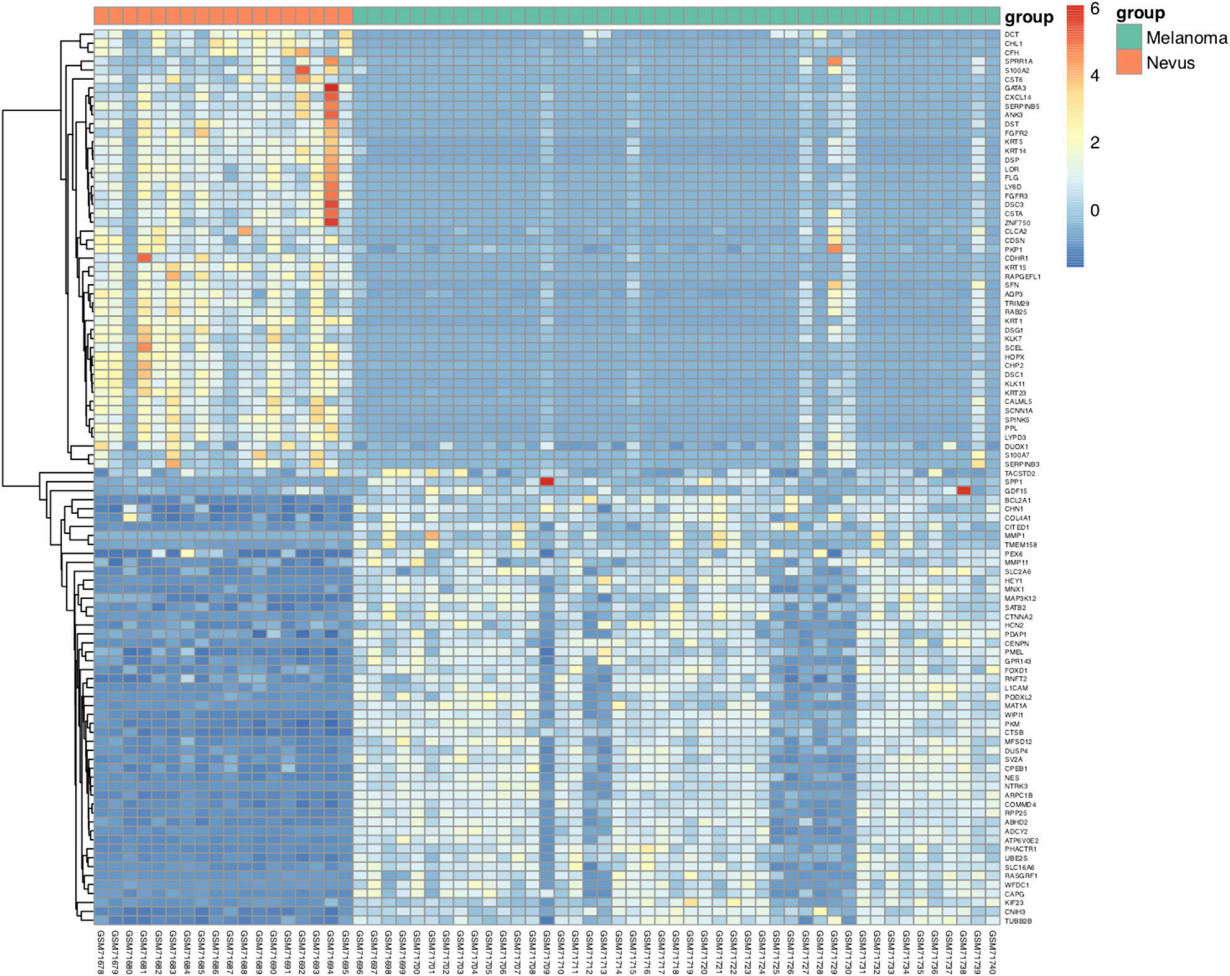
Representative heat map of the top 100 differentially expressed genes indataset GSE3189 (50 up-regulated genes and 50 down-regulated genes). *Red:* upregulation; *blue:* down-regulation.

GEO2R was adopted to identify the DEGs and DMGs, respectively. For DEGs of gene expression microarray, 554 overlapping up-regulated genes (1,088 in GSE3189, 4,096 in GSE114445) and 462 overlapping down-regulated genes (1,224 in GSE3189, 4,152 in GSE114445) were screened. For DMGs of gene methylation microarray, 15,052 overlapping hypermethylation genes (17,016 in GSE86355, 22,767 in GSE120878) and 17,888 overlapping hypomethylation genes (18,944 in GSE86355, 25,934 in GSE120878) were found. As shown in Figure 1, we identified 237 hypomethylated, upregulated genes and 182 hypermethylated, downregulated genes after integrating the DEGs and DMGs. The flowchart was illustrated in Figure 2. The representative heat map of the MeDEGs of GSE3189 (including the top 50 up-regulated and down-regulated genes) was present in Figure 3.

### Functional enrichment analysis of methylation-regulated differentially expressed genes

The results of the GO enrichment analysis for the MeDEGs were shown in Tables 1, 2. For hypomethylation-upregulated genes, changes in biologic processes were mostly enriched in the oxidation-reduction process, cell proliferation, cell division, phosphorylation, extracellular matrix disassembly and protein sumoylation. The hypermethylation-downregulated genes were mainly enriched in positive regulation of transcription from RNA polymerase II promoter, cell adhesion, cell proliferation, positive regulation of transcription, DNA-templated and angiogenesis. We also found that the hypomethylated, upregulated genes were related to cytosol, extracellular exosome, membrane and nucleoplasm, while hypermethylated, downregulated genes to cytoplasm, plasma membrane, cytosol and cell junction in the cellular component group. For hypomethylated, upregulated genes, changes in molecular function were primarily enriched in protein binding, ATP binding, enzyme binding and GTPase activity, and for hypermethylated, downregulated genes, changes were significantly enriched in protein binding, transcriptional activator activity, RNA polymerase II core promoter proximal region sequence-specific binding and transcription factor activity, sequence-specific DNA binding. Pathway enrichment was also performed using KEGG, and the results were shown in Table 3. We found that hypomethylated genes predominantly participated in selenocompound metabolism, small cell lung cancer and lysosome. For hypermethylated genes, the most significantly enriched pathways involved the transcriptional misregulation in cancer, circadian rhythm, tight junction, protein digestion and absorption and Hippo signaling pathway.

**TABLE 1:** Gene ontology enrichment analysis of hypomethylated upregulated genes.

Category	Term	Description	Count	*p*.Value
BP	GO:0001887	selenium compound metabolic process	3	1.68E-03
BP	GO:0016310	phosphorylation	7	2.07E-03
BP	GO:0022617	extracellular matrix disassembly	6	3.27E-03
BP	GO:0000059	protein import into nucleus, docking	3	4.58E-03
BP	GO:0060236	regulation of mitotic spindle organization	3	4.58E-03
BP	GO:0006606	protein import into nucleus	5	7.11E-03
BP	GO:0016925	protein sumoylation	6	1.93E-02
BP	GO:0008283	cell proliferation	11	2.33E-02
BP	GO:0051301	cell division	10	4.25E-02
BP	GO:0055114	oxidation-reduction process	14	4.98E-02
CC	GO:0016020	membrane	59	2.73E-08
CC	GO:0070062	extracellular exosome	67	1.96E-07
CC	GO:0005829	cytosol	75	2.01E-07
CC	GO:0031012	extracellular matrix	15	2.53E-05
CC	GO:0005654	nucleoplasm	54	1.16E-03
CC	GO:0005783	endoplasmic reticulum	22	1.90E-03
CC	GO:0031965	nuclear membrane	10	2.60E-03
CC	GO:0005925	focal adhesion	13	4.18E-03
CC	GO:0001772	immunological synapse	4	8.90E-03
CC	GO:0042470	melanosome	6	9.11E-03
CC	GO:0043231	intracellular membrane-bounded organelle	15	1.16E-02
CC	GO:0005789	endoplasmic reticulum membrane	19	2.54E-02
CC	GO:0005737	cytoplasm	79	4.60E-02
CC	GO:0005794	Golgi apparatus	18	4.67E-02
CC	GO:0005739	mitochondrion	25	4.91E-02
MF	GO:0005515	protein binding	149	7.15E-05
MF	GO:0008139	nuclear localization sequence binding	4	6.28E-03
MF	GO:0005524	ATP binding	32	1.15E-02
MF	GO:0003924	GTPase activity	9	1.47E-02
MF	GO:0019899	enzyme binding	11	1.58E-02
MF	GO:0051015	actin filament binding	6	3.40E-02

**TABLE 2:** Gene ontology enrichment analysis of hypermethylated downregulated genes.

Category	Term	Description	Count	*p*.Value
BP	GO:0007155	cell adhesion	15	2.30E-04
BP	GO:0001954	positive regulation of cell-matrix adhesion	4	1.34E-03
BP	GO:0045944	positive regulation of transcription from RNA polymerase II promoter	21	2.07E-03
BP	GO:0050680	negative regulation of epithelial cell proliferation	5	2.42E-03
BP	GO:0006366	transcription from RNA polymerase II promoter	13	5.80E-03
BP	GO:0090162	establishment of epithelial cell polarity	3	6.22E-03
BP	GO:0001942	hair follicle development	4	6.52E-03
BP	GO:0001525	angiogenesis	8	7.46E-03
BP	GO:0008284	positive regulation of cell proliferation	12	7.63E-03
BP	GO:0016477	cell migration	7	7.97E-03
BP	GO:0008285	negative regulation of cell proliferation	10	1.90E-02
BP	GO:0090090	negative regulation of canonical Wnt signaling pathway	6	2.46E-02
BP	GO:0008283	cell proliferation	9	3.21E-02
BP	GO:0042493	response to drug	8	3.44E-02
BP	GO:0045893	positive regulation of transcription, DNA-templated	11	3.57E-02
CC	GO:0016324	apical plasma membrane	13	3.17E-05
CC	GO:0005911	cell-cell junction	8	1.57E-03
CC	GO:0005737	cytoplasm	70	2.63E-03
CC	GO:0005856	cytoskeleton	10	1.12E-02
CC	GO:0005829	cytosol	46	1.22E-02
CC	GO:0030054	cell junction	11	1.55E-02
CC	GO:0031012	extracellular matrix	8	2.73E-02
CC	GO:0005886	plasma membrane	52	3.85E-02
CC	GO:0005925	focal adhesion	9	3.96E-02
MF	GO:0001077	transcriptional activator activity, RNA polymerase II core promoter proximal region sequence-specific binding	10	5.79E-04
MF	GO:0005515	protein binding	106	2.94E-03
MF	GO:0000989	transcription factor activity, transcription factor binding	3	8.19E-03
MF	GO:0008013	beta-catenin binding	5	8.89E-03
MF	GO:0043565	sequence-specific DNA binding	12	1.41E-02
MF	GO:0003700	transcription factor activity, sequence-specific DNA binding	17	2.88E-02

**TABLE 3:** Pathway enrichment analysis of MeDEGs.

Category	Term	Count	*p*.Value
Hypomethylated upregulated genes			
KEGG_PATHWAY	hsa00450:Selenocompound metabolism	4	2.42E-03
KEGG_PATHWAY	hsa05222:Small cell lung cancer	6	1.22E-02
KEGG_PATHWAY	hsa04142:Lysosome	6	4.70E-02
Hypermethylated downregulated genes			
KEGG_PATHWAY	hsa05202:Transcriptional misregulation in cancer	6	3.51E-02
KEGG_PATHWAY	hsa04710:Circadian rhythm	3	4.45E-02
KEGG_PATHWAY	hsa04530:Tight junction	4	6.89E-02
KEGG_PATHWAY	hsa04974:Protein digestion and absorption	4	7.08E-02
KEGG_PATHWAY	hsa04390:Hippo signaling pathway	5	8.21E-02

### Protein-protein interaction network establishment and hub genes selection

**FIGURE 4:**
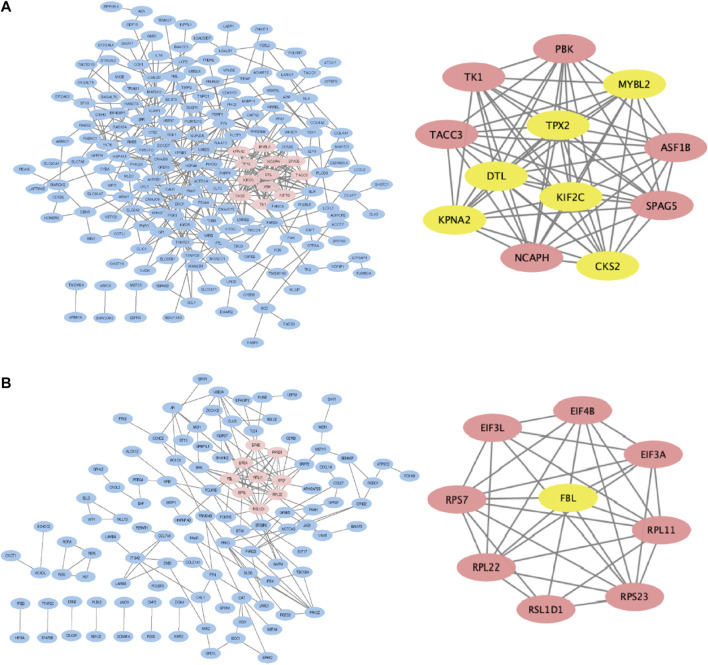
PPI network and most related modules of methylation-regulated differentiallyexpressed genes. **(A)** Hypomethylated upregulated genes. **(B)** Hypermethylated downregulated genes.

The PPI network of hypomethylation-upregulated genes and hypermethylation-downregulated genes was visualized by Cytoscape (version 3.6.0) (Smoot et al., 2011). MCODE (version 1.4.2) is a plug-in of Cytoscape to cluster a given network to select densely connected regions based on topology (Bandettini et al., 2012). The results were presented in Figure 4. Therefore, PBK, TK1, TACC3, MYBL2, TPX2, DTL, KPNA2, KIF2C, CKS2, ASF1B, SPAG5 and NCAPH were verified as hub genes in hypomethylation-upregulated genes module. And EIF4B, EIF3L, EIF3A, RPS7, RPL22, RSL1D1, RPS23, RPL11 and FBL were selected as hub genes in the hypermethylation-downregulated genes module.

### Survival analysis

**FIGURE 5:**
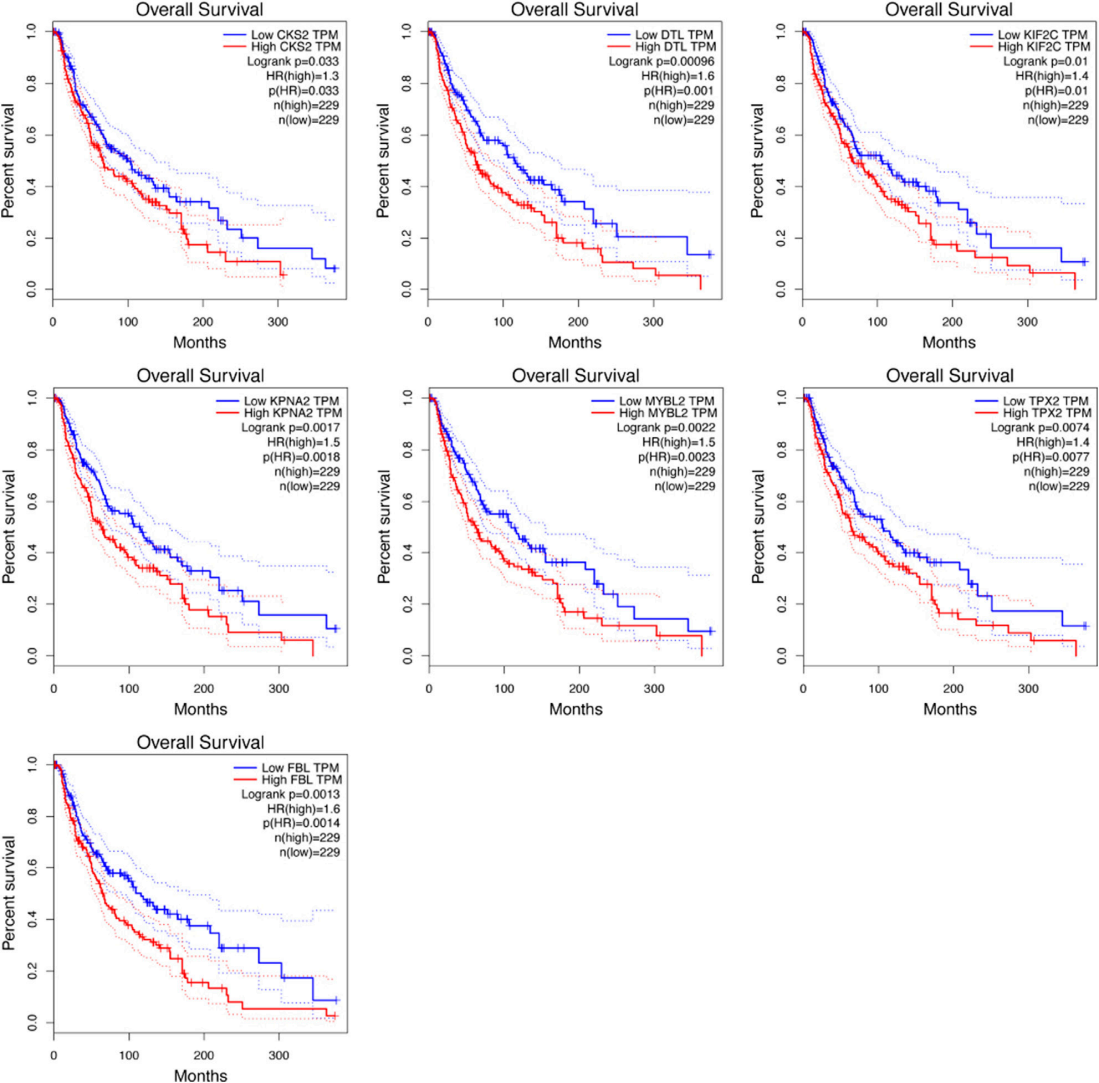
Survival analysis of the hub genes was performed using Kaplan-Meier curve. Each elevated expression in seven hub genes showed markedly significant worse OS inmelanoma samples (*p* < 0.05).

Significant survival outcomes of hub genes in the PPI network were displayed in Figure 5. According to the expression of each gene, overall survival for SKCM patients was acquired. We found that high mRNA expression of CKS2 (*p* = 0.033) was closely related to worse prognosis for SKCM as well as DTL (*p* = 0.00096), KIF2C (*p* = 0.01), KPNA2 (*p* = 0.0017), MYBL2 (*p* = 0.0022), TPX2 (*p* = 0.0074), FBL (*p* = 0.0013).

### Hub genes verification

**FIGURE 6:**
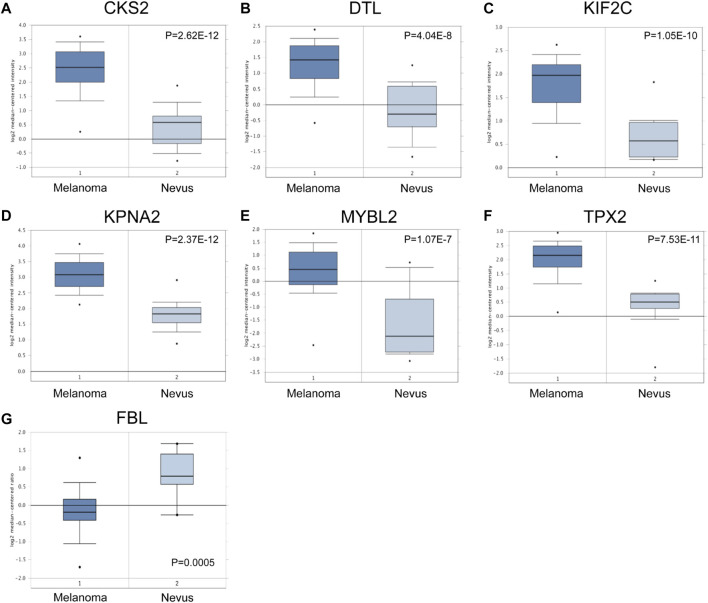
Validation of the expression of hub genes in Oncomine database. Theexpression level of *CKS2, DTL, KIF2C, KPNA2, MYBL2, TPX2* and *FBL* was detected in Oncomine database.

Subsequently, we used the Oncomine database to further validate the expression of seven hub genes. The different expression levels of six hypomethylated upregulated hub genes and one hypermethylated downregulated hub genes between melanoma and nevus samples were significantly obvious (Figure 6), which were consistent with the results we obtained.

**FIGURE 7:**
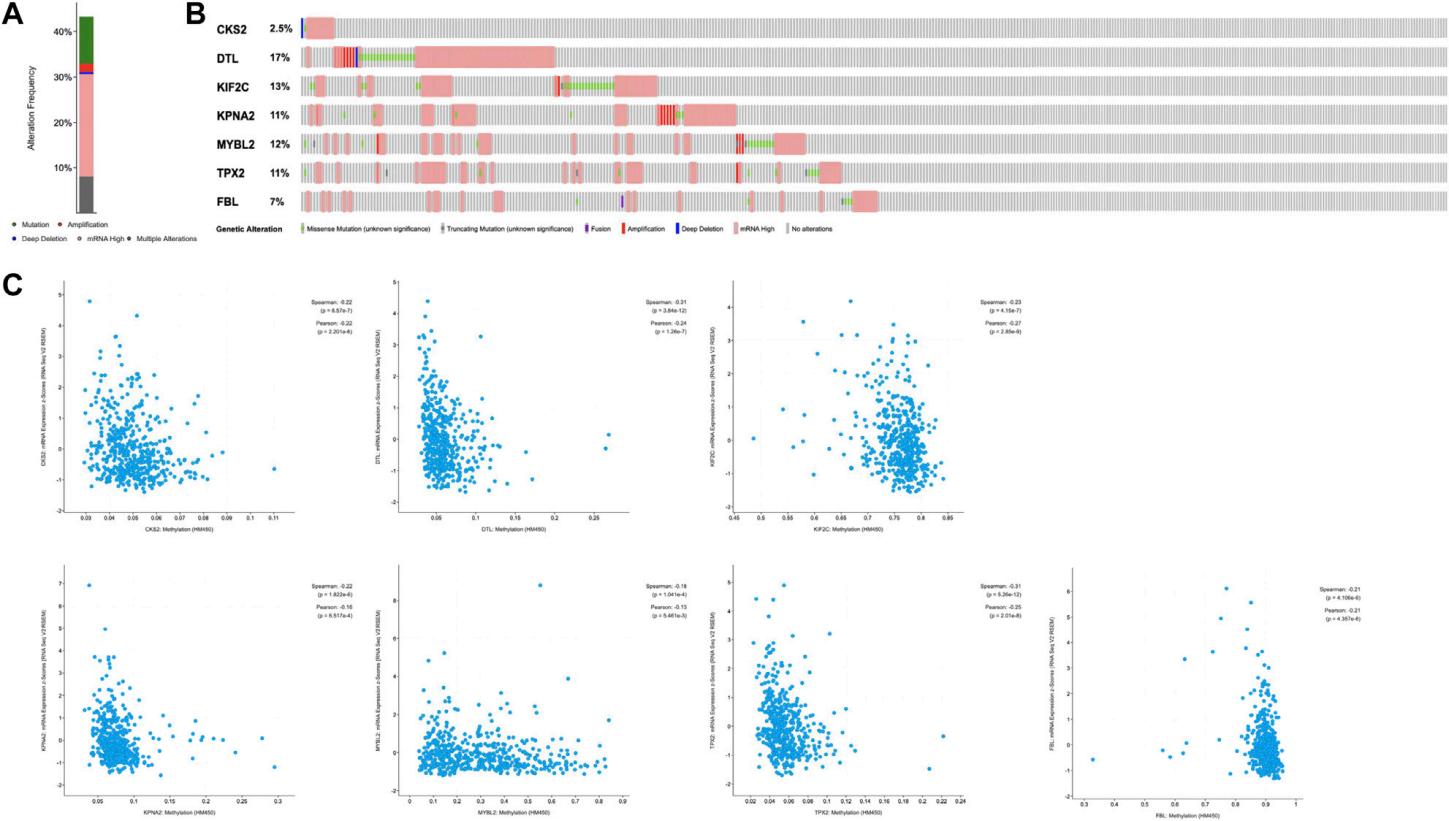
Genetic alteration of seven hub genes and the relationship between mRNAexpression and DNA methylation in the TCGA SKCM study using the cBioPortaldatabase. **(A)** Alteration frequency of hub genes. **(B)** A visual summary of alteration based on a query of seven hub genes, which was altered in 192 (43.24%) of 444 sequenced cases/patients. **(C)** The relationship between mRNA expression and DNA methylation in the seven hub genes.

In addition, we used the cBioPortal tool to explore the genetic alterations of seven hub genes and discovered that DTL (17%) and KIF2C (13%) were the most frequently altered genes among the seven hub genes, including amplification, fusion, and missense mutations (Figures 7A,B). The alterations of the seven hub genes were 192 (43.24%) of 444 sequenced cases/patients. The correlations between mRNA and DNA methylation of the seven genes in the TCGA SKCM patients were demonstrated in Figure 7C. We found that the correlation was negative, indicating that methylation regulated the mRNA expression of these genes. This illustrated that methylation played an important role in the expression of these genes.

### Significant genes and pathways obtained by gene set enrichment analysis

**FIGURE 8:**
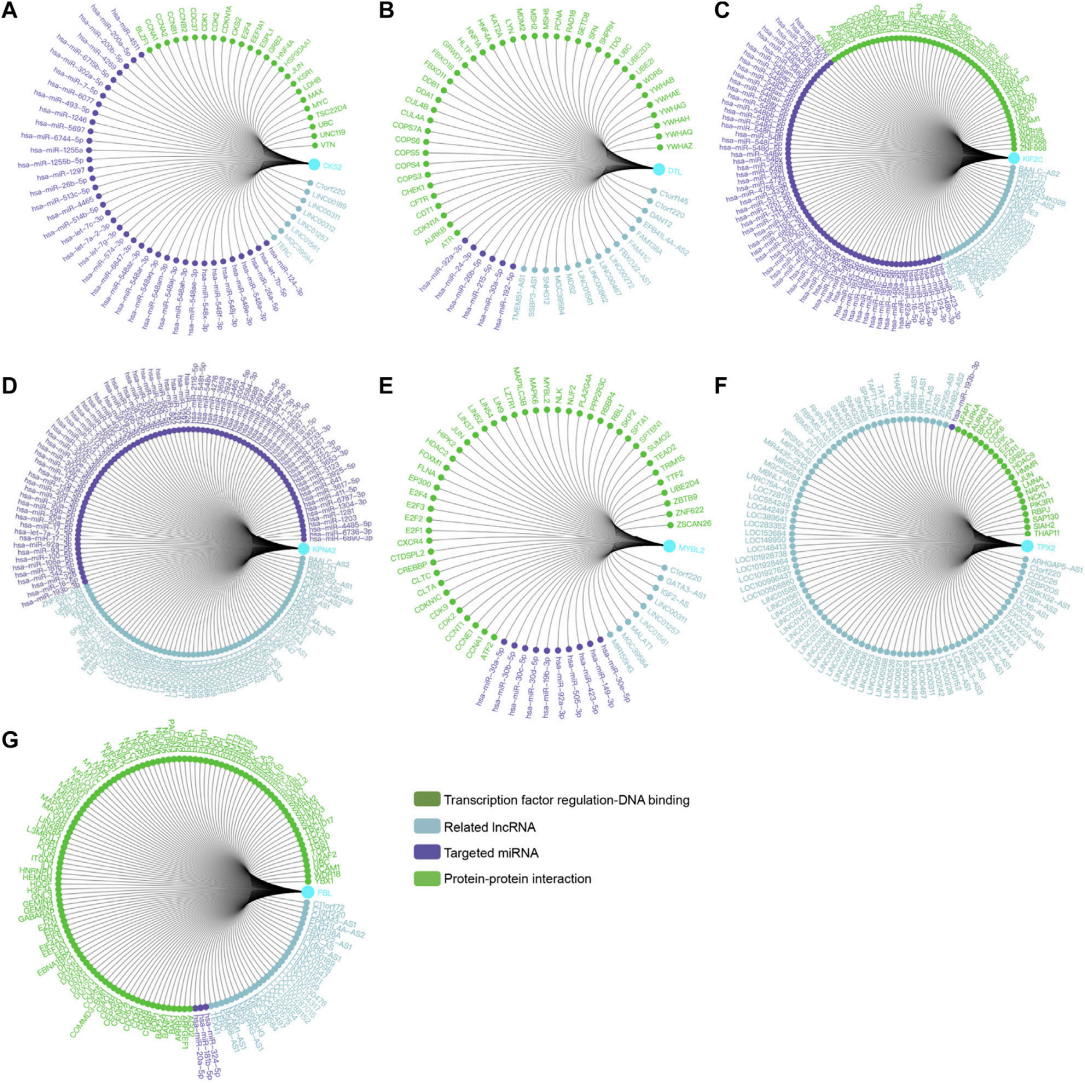
Transcription factor regulation network was constructed in *CKS2, DTL, KIF2C, KPNA2, MYBL2, TPX2,* and *FBL*. Significant nodes were marked in different colors in line with hub genes (Transcription factor regulation-DNA binding, Related lncRNA, Targeted miRNA and Protein-protein interaction).

**FIGURE 9:**
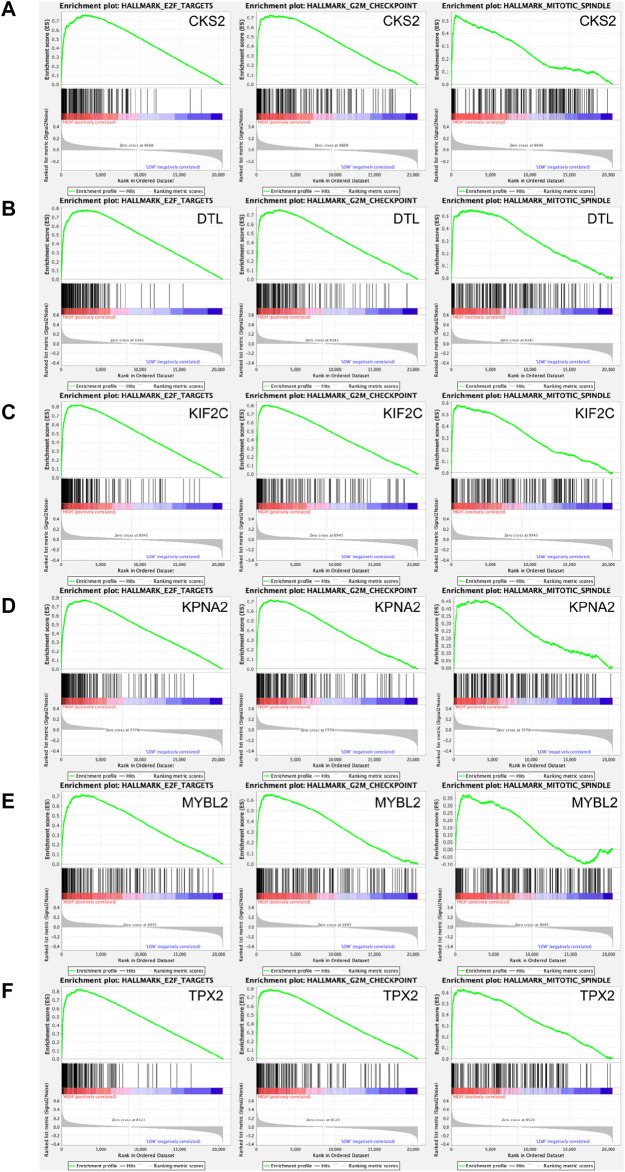
A total of 100 significant genes were obtained from GSEA with positive and negative correlations. GSEA was used to perform hallmark analyses in *CKS2, DTL, KIF2C, KPNA2, MYBL2* and *TPX2*, respectively. The most involved significant pathways included E2F targets, G2M checkpoint and mitotic spindle.

Transcriptional regulation networks among *CKS2, DTL, KIF2C, KPNA2, MYBL2, TPX2* and *FBL* were displayed in Figure 8. Significantly involved nodes (including transcription factor regulation-DNA binding, related lncRNA, targeted miRNA and protein-protein interaction) were marked in different colors. Subsequently, a total of 100 significant genes were obtained from GSEA, and the genes with positive correlations were plotted. GSEA analysis, including *CKS2, DTL, KIF2C, KPNA2, MYBL2,* and *TPX2* indicated that the most involved hallmarks pathways were E2F targets, G2M checkpoint and mitotic spindle. The details were illustrated in Figure 9.

### Kinesin family member 2C expression pattern and its pro-tumorigenic malignant biological behaviors in skin cutaneous melanoma

To reveal levels of expression and methylation of KIF2C in SKCM samples, we first enrolled 33 SKCM tissues and assessed the protein expression of KIF2C in SKCM using IHC analysis (Supplementary Figure S1A). Methylation-specific PCR was used to detect the methylation expression of KIF2C. Pearson correlation analysis suggested showed a significantly negative association between the protein and methylation expression (r = −0.362, *p* < 0.001) (Supplementary Figure S1B). Then, qRT-PCR was used to detect the knockdown efficacy of siRNAs silencing the expression of KIF2C in A375 cells. Significantly, compared with the negative control group, transcriptional expression of KIF2C was decreased in siRNA1- and siRNA2-treated groups. Therefore, siRNA1 and siRNA2 were used for further experiments (Supplementary Figure S1C). According to the results of the CCK-8 assay, the down-regulated level of KIF2C expression significantly inhibited the proliferative ability of SKCM cells (Supplementary Figure S1D). Transwell cell migration assay indicated that the down-expression of KIF2C suppressed the metastasis capacity of SKCM cells (Supplementary Figure S1E).

## Discussion

Melanoma is an aggressive and devastating cancer that can be directly derived from melanocytic nevus. Nowadays, surgery of tumor resection before metastasis still remains the most effective treatment (Gadeliya Goodson and Grossman, 2009). Thus, it is important to diagnose the high-risk nevus in the early stage. Identification of novel biomarkers will be greatly helpful to improve diagnosis and an even better understanding of the mechanism involved in melanocytic tumorigenesis that potentially contributes to novel therapy. Hence, highly effective biomarkers for diagnosis and treatment are urgently required.

The initiation and progression involved in melanoma is a complicated and multistage process regulated by both genetic and epigenetic alterations. Increasing evidence has shown the essential roles of epigenetic modifications, especially DNA methylation, in SKCM (Schinke et al., 2010; Berger et al., 2012; Goran Micevic et al., 2017). However, most of these studies are limited to melanoma metastases and lack primary melanomas, which made it difficult to identify early biological progress during melanoma development (Weiyang Cai et al., 2018). Furthermore, separate analyses of gene expression and methylation from one study are limited (Wouters et al., 2017), while integrating multiple available datasets may help us find more accurate and reliable evidence through comprehensive bioinformatics analysis. Yet, conjoint analysis including both gene expression and methylation profiling microarray datasets is largely insufficient in SKCM. Therefore, we conducted an integrated bioinformatics analysis based on both gene expression and gene methylation profiling to identify the new prognostic biomarkers and therapeutic targets in SKCM for future research.

In the present study, we identified a total of 237 hypomethylated, upregulated genes and 182 hypermethylated, downregulated genes by overlapping the DEGs and DMGs. For the hypomethylation-upregulated genes, functional enrichment analysis indicated that changes in the biological processes were mainly enriched in the oxidation-reduction process, cell proliferation, cell division, phosphorylation, extracellular matrix disassembly and protein sumoylation. GO cell component analysis showed that the upregulated genes were significantly enriched in cytosol, extracellular exosome, membrane and nucleoplasm. In addition, for molecular function, the hypomethylation-upregulated genes were significantly enriched in protein binding, ATP binding, enzyme binding and GTPase activity. KEGG pathway enrichment analysis suggested significant enrichment in pathways including selenocompound metabolism, small cell lung cancer and lysosome. Notably, GSEA results showed that E2F targets, G2M checkpoint and mitotic spindle were the most involved hallmarks in SKCM. These findings are reasonable because it is universally acknowledged that the above processes are closely related to tumor initiation and progression, including melanoma (Hanahan and Weinberg Robert, 2011).

PPI network of hypomethylation-high expression genes illustrated the protein-protein interactome of the hub genes, and then GEPIA was adopted to select the most prognostic hub genes, named *CKS2, DTL, KIF2C, KPNA2, MYBL2* and *TPX2*, which may provide new clues for the therapeutic strategy in SKCM.

Cyclin-dependent kinases regulatory subunit 2 (CKS2), a cyclin-dependent kinase-interacting protein, is critical for cell cycle regulation. Overexpression of CKS2 has been reported to be associated with several types of cancer, including colorectal cancer and cervical cancer (Yu et al., 2015; Jonsson et al., 2019). de Wit et al. (2005) reported that CKS2 could be considered a candidate player in melanocytic tumor progression and facilitate early diagnosis of melanocytic lesions before metastasis. We found that the expression of CKS2 in SKCM tissues was higher in different datasets, and survival analysis revealed that the upregulation of CKS2 was related to a worse prognosis in SKCM, which was consistent with the results of previous studies.

Denticleless E3 ubiquitin protein ligase homologue (DTL), also known as DNA replication factor 2, can regulate the expression of various cell cycle regulatory proteins and maintain the integrity of DNA replication and repair (Pan et al., 2006). Elevated expression of DTL has been found to be related to a variety of cancers, such as breast cancer, Ewing sarcoma and ovarian cancer (Ueki et al., 2008; Mackintosh et al., 2012; Pan et al., 2013). Yang et al. (2019) suggested that DTL can be regarded as an indicator of poor prognosis in acral melanoma patients. DTL could play an important role in promoting melanoma cell growth and glucose metabolism, possibly through activation of the MYC target pathway (Lu et al., 2022). In the present study, we found that overexpressed DTL was closely associated with worse survival outcomes in cutaneous melanoma patients. The rate of DTL mutation was 17%, and this higher mutation rate may lead to abnormal methylation or deregulation of DTL.

As a member of the kinesin-13 family, kinesin family member 2C (KIF2C) uses microtubule depolymerizing activity to correct improper microtubule attachments at kinetochores, which plays significant roles during the mitosis process (Lawrence et al., 2004; Gardner Melissa et al., 2011). KIF2C is likely to be the essential gene for carcinogenesis and may be closely involved in tumor-infiltrating lymphocytes of cancer immunotherapy for patients with metastatic melanoma (Lu et al., 2014). In our study, we found that the expression of KIF2C was significantly elevated in SKCM tissues compared to nevus tissues and associated with poor prognosis in melanoma patients by bioinformatic research. Importantly, we validated that the down-regulated level of KIF2C expression significantly inhibited the proliferative ability and suppressed the metastasis capacity of SKCM cells.

*KPNA2,* a member of karyopherin (KPNA) protein family, is considered as a key role in the malignant transformation of cells through the transport of tumor suppressors, regulation of DNA repair proteins as well as activation of apoptosis pathways (Tseng et al., 2005). Elevated KPNA levels have been found to predict poor prognosis for multiple tumors, including breast and cervical cancer (Dahl et al., 2006; van der Watt et al., 2009). Winnepenninckx et al. (2006) reported that KPNA2 is closely associated with poor prognosis and tumor progression in melanoma, which is consistent with our results. Yang et al. (2020) found that KPNA2 promotes proliferation, invasion and migration through NF-κB/p65 signaling pathways in melanoma cells. In this study, we suggested the possibility of the aberrant methylation of the KPNA2 promoter.

Myb proto-oncogene like 2 (MYBL2*),* located on chromosome 20q13, acts as a transcription factor that plays a significant role in cell-cycle progression. In the previous study, overexpression of MYBL2 has been found to be related to poor prognosis in various cancers, such as prostate and gallbladder cancer (Bar-Shira et al., 2002; Liang et al., 2017). Koynovaa et al. (2007) found a higher frequency of low-level increase of the copy numbers of MYBL2 rather than amplification in melanoma. Also, evidence showed that attenuation of miR-29b2∼c expression promotes the development of melanoma by partly depressing MAFG and MYBL2 (Vera et al., 2021). Taken together, this evidence indicated that MYBL2 was involved in cell proliferation and tumorigenesis in melanoma, which is consistent with our present findings. However, further research is needed to confirm our hypothesis.

Targeting protein for xenopus kinesin-like protein 2 (TPX2, also known as REPP86) located on chromosome 20q11.2 in humans. TPX2 is a mitotic microtubule-associated protein that is strictly regulated by the cell cycle and diffusely distributed during the S and G2 phases, which help spindle stability (Alfaro-Aco and Petry, 2017). Ping Wei et al. (2013) demonstrated that upregulation of TPX2 was associated with the clinical stage, invasion and metastasis in colon cancer, participating in the P13K/Akt signaling pathway to reduce the occurrence as well as the proliferation of colon cancer cells. Increased expression of TPX2 was also observed in lung squamous cell carcinoma, ovarian cancer and giant-cell tumor of the bone (Ignacio Blanco et al., 2015; Laura et al., 112006; Aguirre-Portoles et al., 2012). Yao et al. (2018) showed that TPX2 improved the proliferative ability of melanoma cell lines and functioned as an oncogene in melanoma. In our study, TPX2 was found to be a hypomethylated-upregulated gene in melanoma and associated with the poor prognostic of melanoma patients, which suggested that TPX2 may be used as a novel prognostic marker for the development and progression of SKCM. Our results were consistent with the roles of TPX2 in various tumors reported in previous studies.

As for the hypermethylation-downregulated genes, functional enrichment analysis indicated that changes in biological processes were mainly enriched in the positive regulation of transcription from RNA polymerase II promoter, cell adhesion, cell proliferation, positive regulation of transcription, DNA-templated and angiogenesis. GO cell component analysis showed that the downregulated genes were significantly enriched in the cytoplasm, plasma membrane, cytosol and cell junction. Besides, for molecular function, the hypermethylation-downregulated genes were significantly enriched in protein binding, transcriptional activator activity, RNA polymerase II core promoter proximal region sequence-specific binding and transcription factor activity, and sequence-specific DNA binding. KEGG pathway enrichment analysis suggested significant enrichment in pathways including transcriptional misregulation in cancer, circadian rhythm, tight junction, protein digestion and absorption and Hippo signaling pathway. Importantly, growing evidence showed that numerous genetic changes in melanoma may be linked to in Hippo signaling pathway (Kim et al., 2013; Feng et al., 2017). Moreover, Hippo pathway was found to be correlated with the mitogen-activated protein kinase (MAPK) signaling pathway which is well known for a vital role in the pathogenesis of melanoma (Feng et al., 2017).

Then, we performed a PPI network and survival analysis to identify the prognostic hub gene among the hypermethylation-downregulated genes. Fibrillarin (FBL) is an indispensable, highly conserved protein essential in the processing of pre-rRNAs (Newton et al., 2003). In the previous study, the expression of FBL can be regulated by p53 in multiple tumors and was considered as an ideal target to inhibit the ribosome biogenesis process in cancer therapy (Marcel et al., 2013; El Hassouni et al., 2019). Overexpression of FBL was found in breast, prostate cancers and squamous cell cervical carcinoma (Choi et al., 2007; Koh et al., 2011; Su et al., 2014). Thus, FBL could have a role in tumor progression and could affect the clinical outcome of patients through alteration of translational regulation in melanoma. In our study, we found that the low expression of FBL in SKCM tissues compared to nevus tissues was observed in multiple datasets, and survival analysis showed that the high expression of FBL was related to a worse prognosis in SKCM.

The present study constructed a comprehensive network between nevus and melanoma and identify the prognostic significance of these hub genes, which may serve as valuable prognostic indicators of SKCM. However, there are still some limitations in this study: firstly, the gene expressions and methylation profiles were from different studies (Wu et al., 2018); secondly, the survival outcomes might be heavily contaminated due to cancer subtypes (Ren et al., 2019), thus the result could be not very stable. Furthermore in-depth investigation through *in vivo* and *in vitro* experimental designs and analyses are needed in future work.

## Conclusion

In summary, this study identified methylation-regulated differentially expressed genes and related pathways and functions in SKCM by using integrated bioinformatics analysis. In addition, we constructed PPI networks and performed survival analysis that identified seven prognostic hub genes. Our findings may deepen the understanding of the methylation-mediated regulatory mechanisms underlying the carcinogenesis possibility of melanocytic nevus and melanoma and provide novel biomarkers and therapeutic targets for further research.

## Supplementary material

Supplementary Figure S1 Validation of the KIF2C role in SKCM. **(A)** IHC analysis was performed to reveal high expression of KIF2C in SKCM samples. **(B)** Methylation specific PCR was used to detect the methylation expression of KIF2C. Pearson correlation analysis suggested showed significantly negative association between the protein and methylation expression (r = −0.362, *p* < 0.001). **(C)** qRT-PCR was used to detect the knockdown efficacy of siRNAs silencing expression of KIF2C in A375 cells. Significantly, compared with the negative control group, transcriptional expression of KIF2C was decreased in siRNA1- and siRNA2-treated groups. **(D)** According to the results of the CCK-8 assay, down-regulated level of KIF2C expression significantly inhibited the proliferative ability of SKCM cells. **(E)** Transwell cell migration assay indicated that the down-expression of KIF2C suppressed metastasis capacity of SKCM cells.

## Abbreviations

SKCM, skin cutaneous melanoma; DEGs, differentially expressed genes; DMGs, differentially methylated genes; MeDEGs, methylation-regulated differentially expressed genes; DAVID, the Database for Annotation, Visualization and Integrated Discovery; GEPIA, Gene Expression Profiling Interactive Analysis; GO, gene ontology; BP, biological process; CC, cellular component; MF, molecular function; KEGG, Kyoto Encyclopedia of Genes and Genomes; PPI, protein-protein interaction; TCGA, the Cancer Genome Atlas; GTEx, Genotype-Tissue Expression project; OS, overall survival; GSEA, gene set enrichment analysis; MARK, mitogen-activated protein kinase.
