# Supplementary material for: Identification of aberrantly methylated differentially expressed genes and pro-tumorigenic role of KIF2C in melanoma
Source: Front Genet. 2022 Jul 22;13:817656. doi: 10.3389/fgene.2022.817656 (PMC9387026; doi:10.3389/fgene.2022.817656)
Supplement: Supplementary file 1 [file Table1.docx]

| **Primer** | **Sequence( 5’- 3’)** |
| --- | --- |
| KIF2C-F | GATGGAAGCCTGCTCTAACG |
| KIF2C-R | GAGCAGATTCCGCTTTGTTC |
| β-actin-F | CATTAAGGAGAAGCTGTGCT |
| β-actin-R | GTTGAAGGTAGTTTCGTGGA |

**Table S1. The primer sequences for KIF2C and β-actin.**
