# Supplementary figures and images for: Identification of aberrantly methylated differentially expressed genes and pro-tumorigenic role of KIF2C in melanoma
Source: Front Genet. 2022 Jul 22;13:817656. doi: 10.3389/fgene.2022.817656 (PMC9387026; doi:10.3389/fgene.2022.817656)

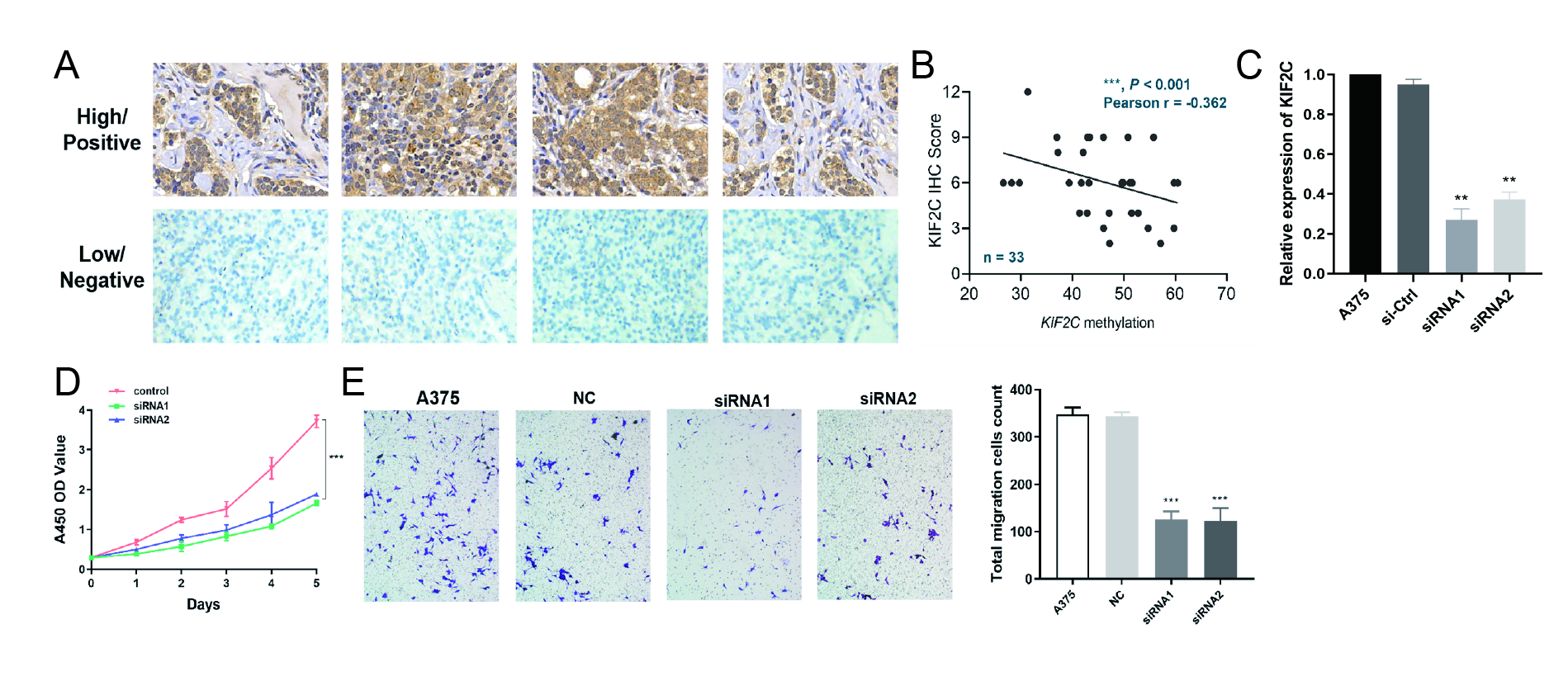

Supplement: Supplementary file 2 [file Image1.tif]

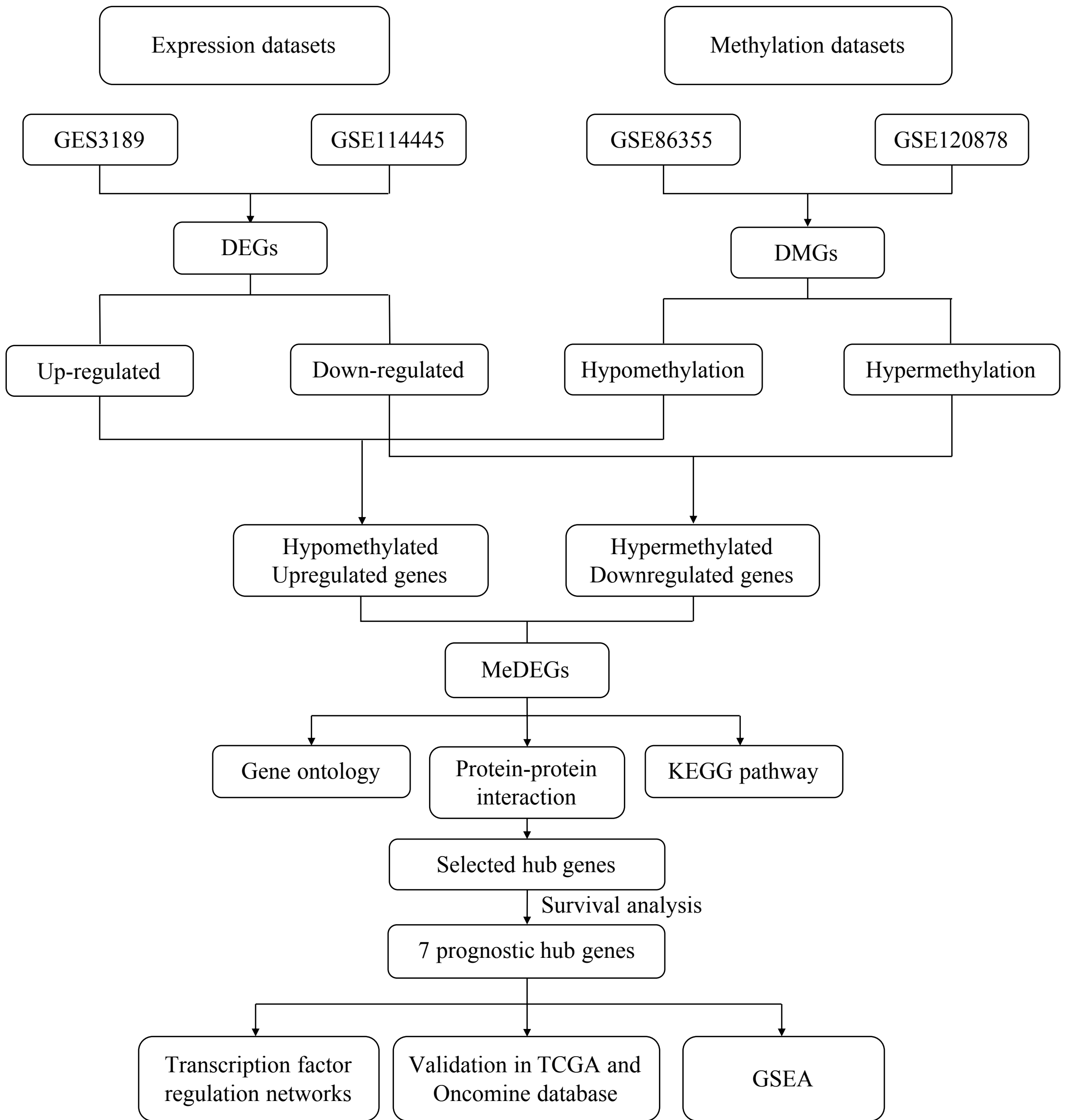

Supplement: Supplementary file 3 [file DataSheet1.PDF]
